# Using the scenario method in the context of health and health care – a scoping review

**DOI:** 10.1186/s12874-015-0083-1

**Published:** 2015-10-16

**Authors:** Horst Christian Vollmar, Thomas Ostermann, Marcus Redaèlli

**Affiliations:** 1 Institute of General Practice, Medical Faculty, Heinrich-Heine-University Düsseldorf, Moorenstr. 5, 40225 Düsseldorf, Germany; 2 Institute of General Practice and Family Medicine, Faculty of Health, Witten/Herdecke University, Witten, Germany; 3 Institute of Integrative Medicine, Faculty of Health, Witten/Herdecke University, Witten, Germany; 4 Institute of Health Economics and Clinical Epidemiology, University Hospital Cologne, Cologne, Germany

**Keywords:** Scenario method, Health planning, Public health administration, Planning techniques, Decision-making, Policy making, Foresight, Forecasting

## Abstract

**Background:**

The scenario technique is a method for future research and for strategic planning. Today, it includes both qualitative and quantitative elements. The aims of this scoping review are to give an overview of the application of the scenario method in the fields of health care and to make suggestions for better reporting in future scenario projects.

**Methods:**

Between January 2013 and October 2013 we conducted a systematic search in the databases Medline, Embase, PsycInfo, Eric, The Cochrane Library, Scopus, Web of Science, and Cinahl since inception for the term ‘scenario(s)’ in combination with other terms, e.g. method, model, and technique. Our search was not restricted by date or language. In addition, we screened the reference lists of the included articles.

**Results:**

A total of 576 bibliographical records were screened. After removing duplicates and three rounds of screening, 41 articles covering 38 different scenario projects were included for the final analysis. Nine of the included articles addressed disease related issues, led by mental health and dementia (*n* = 4), and followed by cancer (*n* = 3). Five scenario projects focused on public health issues at an organizational level and five focused on the labor market for different health care professionals. In addition, four projects dealt with health care ‘in general’, four with the field of biotechnology and personalized medicine, and additional four with other technology developments. Some of the scenario projects suffered from poor reporting of methodological aspects.

**Conclusions:**

Despite its potential, use of the scenario method seems to be published rarely in comparison to other methods such as the Delphi-technique, at least in the field of health care. This might be due to the complexity of the methodological approach. Individual project methods and activities vary widely and are poorly reported. Improved criteria are required for reporting of scenario project methods. With improved standards and greater transparency, the scenario method will be a good tool for scientific health care planning and strategic decision-making in public health.

**Electronic supplementary material:**

The online version of this article (doi:10.1186/s12874-015-0083-1) contains supplementary material, which is available to authorized users.

## Background

Strategic decision-making processes in the field of health care and public health have always been a point of critical discussion between the stakeholders involved. In particular, prospective planning of financial resources for epidemiologically relevant and cost intensive diseases, like dementia, is often challenging. In such cases tools to support stakeholders in the field of evidence-based decision-making have become quite important [1]. Tools often used in strategic decision-making in public health are consensus processes e.g. the Delphi technique [2, 3]. These approaches use rounds of questionnaire surveys where information and results are fed back to panel members between each round [4]. According to a recent review by Diamond and colleagues, 98 % of Delphi studies claimed to assess consensus [5]. Other methods used by policy makers in the provision of health care are based on simulation modeling. In their systematic review of the “use and value of computer simulation modeling in population health and health care delivery”, Fone et al. found 182 papers using simulation techniques in the field of public health [6]. Both the Delphi technique and simulation modeling are widely used for health care issues, but were originally developed to support forecast and foresight processes [7]. These methods are not without critique and leave room for improvement [5, 8].

Over the last decades, the scenario method has become an additional tool in foresight activities and research. To a certain extent, it makes use of both qualitative (e.g. expert opinion and discussion) and quantitative elements (e.g. scenario calculations) [9–11].

Glenn gave the following definition: “a scenario is a story with plausible cause and effect links that connects a future condition with the present, while illustrating key decisions, events, and consequences throughout the narrative” [9]. Scenarios are often described as outlines of possible futures, but they do not describe comprehensive pictures of the future and do not claim to be complete or correct [9, 10, 12, 13]. Although, scenarios are always hypothetical, they are not arbitrary [10]. Additionally, the creation of scenarios presents an interdisciplinary approach to explore future issues while offering several advantages, e.g. the support of a future-oriented way of thinking by taking alternative developments into consideration [9–11]. Furthermore, it fosters systematic and structured discussion of uncertain alternative futures by the incorporation of expert knowledge. Proceeding step-by-step reduces the perceived complexity of the correlations examined, generates findings that are comprehensible [14], and should improve strategic decision-making [15–17]. It may be combined with other foresight methods such as the Delphi technique or road-mapping [18, 19]. Since its first appearance in the 1950s, and after a decline during the 1980s, the number of published articles using this method is again increasing [20].

Although less commonly used in the context of health and health care than either the Delphi technique or simulation modeling, the scenario method has also been used to support strategic decision-making in the field [13, 15–17, 21–25]. Unfortunately, information about the different scenario projects in the context of public health or health policy seems to be disparate and often not known to researchers in this field. Thus, there is a basic need to provide an overview of published scenario projects. The first aim of this review is to give such an overview of the application of the scenario method in the context of health and health care. The second aim is to make first recommendations for improved reporting in future scenario projects.

## Methods

**Fig. 1:**
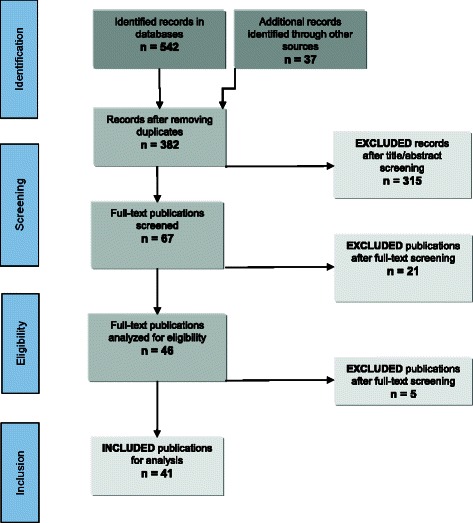
Flow chart of the review process

We conducted our scoping review [26] on the basis of the enhanced recent recommendations of Arksey and O’Malley [27] by Levac and colleagues [28] and presented the results of our search strategy in a flowchart (Fig. 1). Between January 2013 and October 2013 we conducted a systematic search in the following: Medline, Embase, PsycInfo, Eric, The Cochrane Library, Scopus, Web of Science, and CINAHL. An initial search was carried out in January 2013 and an additional search was made in October 2013. The word ‘scenario’ was searched in combination with other terms, e.g. method, model, technique, etc. (see Additional files 1 and 2). In an additional step, the reference lists were tracked backwards for further relevant publications not listed in the databases mentioned above (Fig. 1). We also included in our review manuscripts which were recommended by authors or experts in the field [16, 29]. We also scanned articles suggested by the ´related citations in PubMed’ option for the three most recently published articles [16, 21, 30]. Our search was not restricted by date or language. After screening the title, and, if available, the abstract, all articles that both dealt with the scenario method and addressed issues related to health or health care were included for full text screening. This full screening was performed by two reviewers (HCV, MR) with the following exclusion criteria. Discrepancies were discussed between the two reviewers to achieve consensus. In the case of a possible disagreement a third author (TO) was designated (not required).

Articles were excluded in which the term ‘scenario’ was used only to refer to a possible (future) event [31, 32]. Other exclusion criteria were:

- ‘scenarios’ in epidemiology when used only as projections (e.g. ‘population ageing’, defined as an increase in the percentage of elderly persons in the population) [33],
- ‘scenarios‘which were ‘pure’ simulation modeling [34, 35],
- ‘scenarios’ which were used only to support shared decision-making (e.g. determination of patient preferences) [36, 37],
- ‘scenarios’ in microbiology or genetics [38, 39],
- description of the scenario method itself (without a concrete project) [40–42],
- publications unrelated to health or health care [43],
- abstracts only (no full-text available or full-text already included in our review) [44–46],
- grey literature (e.g. reports) without publication in a scientific journal [25, 47].

## Results

Our search resulted in the identification of a total of 576 bibliographical records. Charting of the data was undertaken independently by two authors (HCV, MR). After removing duplicates, 379 references remained, which, after thorough title and abstract screening, left 67 selected references for possible inclusion (Fig. 1). After full-text screening, 21 publications were excluded because they did not fit the criteria previously determined. From these 46 articles describing the scenario method in use, a total of 41 remained after a final step of exclusion (Fig. 1). Reasons for exclusion were as follows: description of a whole national scenario program (see below) [48], only an abstract of an included project [46], only a simulation modeling project without the description of the scenarios [49], one background paper of an included project with no direct link to the scenarios [50], and one article with no scenario project [51]. The final 41 publications described a total number of 38 different scenario projects. As the studies were quite heterogeneous and included a variety of perspectives, it was decided to classify them by using the following categories (according to Schnaars): year, institution, country, focus, time horizon, number of developed scenarios (Table 1) [52]. We also had discussed to include details of the used methods (e.g. qualitative, quantitative, or both), however in many studies the methods were not adequately described, so we decided not to expand this point. Table 1 gives an overview of the subjects of the included scenario projects, most of them with a time horizon from 10 to 20 years (*n* = 28), two with a 5 year- [13, 53] and one with a 50 year-perspective [54]. In seven projects the adopted time horizon was not mentioned [24, 55–60]. The background of the participating experts was not always adequately reported. The reported background of the experts ranged from “leading health futurists” [61], “members of scientific expert societies and/or staff associations” [55, 62], “experienced managers” [56], “RAND health researchers” [63], “genomic experts and breast cancer specialists” [64], “younger citizens” [65], to “secondary school pupils and university students” [29] or “community members” [30]. Only two projects explicitly stated the background of the experts in a table [16, 66]. Five projects reported a combination of the scenario method with a Delphi technique to reach consensus among participating experts [18, 64, 67–71]. One project was published in Dutch [70] and one in German [55]. All others were published in English. The number of developed scenarios ranged from one [72] to 19 [73] scenarios, but most frequently the numbers of scenarios were three (*n* = 10), four (*n* = 9) or five (*n* = 6) (Table 1).

**Table 1:** Subjects of the scenario projects

Year of publication	Reference	Institution	Country	Focus/title of the project	Time horizon [approx. in years]	Number of scenarios
Before 1995						
2 x 1988	Becker [81], also Schreuder [73]	STG^b^	Netherlands	Aging in the Netherlands	10	3
1991, 1992	Bijl [69] also Bijl & Ketting [70]	STG^b^	Netherlands	Dementia in the Netherlands	10	3
1994	Leufkens et al. [79]	STG^b^	Netherlands	Future of Medicine	10 (15)	4
1988, 1989	Schaapveld & Cleton [74] also Schreuder [73]	STG^b^	Netherlands	Cancer diseases in the Netherlands	15	>5
1988	Schreuder [73]	STG^b^	Netherlands	Cardio-vascular diseases in the Netherlands	15	19
1989, 1997	Van Beeck et al.[68] also van Beeck & Mackenbach [67]	STG^b^	Netherlands	Accident mortality and unintentional injuries in the Netherlands	15	9
1992	Bezold [61]	IAF^a^	US	Leadership practices and organizational demands	10	5
1993	Venable et al. [53]	University of Alabama	US	Local public health departments	5	2
1991	Zentner [76]	Institute for Health Care Marketing	US	Health care organization (case example: future opportunities of American Transitional Care, Inc.)	10	3
1995-2004						
2001	Harmsen et al. [80]	Aarhus School of Business	Denmark	Danish food industry	10	3
1999	Islei et al. [56]	Various universities	UK	Pharmaceutical industry	n.s.^c^	7
1997	Leufkens et al. [60]	Department of Pharmaco-epidemiology & Pharmacotherapy	Netherlands	Clinical pharmacy	n.s.^c^	3
2000	Ling & Hadridge [78]	Cambridge Foresight	UK	Health care	(15-) 20	2
2004	Neiner et al. [24]	National Center for Chronic Disease Prevention and Health Promotion	US	Public health (specifically to illustrate a health department’s desire to address chronic disease prevention and control)	n.s.^c^	3
1998	Nielsen [72]	Allen Memorial Hospital	US	Healthcare delivery	10	1
2001	Sager [59]	Life Science Strategy Consulting	US	Biotechnology	n.s.^c^	4
2003	Van Lente et al. [71]	University of Utrecht	Netherlands	Biotechnology in Europe	10-15	4
Since 2005						
2005	Bezold [22]	IAFa/Picker Institute	US	Patient-centered care	10	4
2005	Bezold & Beck [54]	IAFa	US	Drug regulation	50	3
2011	Bierbooms et al. [13]	Tilburg University	Netherlands	What types of residence should be organized for people with mental health problems?	5	4
2012	Buchan & Seccombe [23]	Queen Margaret University	UK	Future supply of registered nursing staff, midwives and health visitors in the National Health Service (NHS)	10	8
2013	Carlsen et al. [84]	Defense Research Agency	Sweden	Local adaptation to climate change (health aspects)	20	2
2 x 2005	Clark et al. [83] also Awasthi et al. [82]	International Campaign to Revitalise Academic Medicine (ICRAM)	International	Academic medicine	20	5
2006	Eberl & Schnepp [55]	The German Nurses Association	Germany	Family health nursing in Germany	n.s.^c^	7 reduced to 5
2011	Enzmann et al. [62]	Society of Chairs of Academic Radiology Departments (SCARD)	US	Field of radiology	10	3
2012	Gnatzy & Moser [18]	EBS Business School, Deutschland	India (Germany)	Evolving health insurance industry in rural India	10	4
2014	Gregório et al. [16]	WHO Collaborating Centre for Health Workforce Policy and Planning	Portugal	Community pharmacists	10	3
2013	Karger [29]	Forschungs-zentrum Jülich	Germany	Personalized medicine on the example of dementia	20	4
2006	Ma & Seid [63]	RAND cooperation	US	Disease management in the US	15	8
2009	Meristö [57]	My wellbeing project	Finland	Life control especially related to health and personal wellbeing using ICT-tools	n.s.^c^	3
2014	Nguyen et al. [30]	Centre for Public Health and Ecosystem Research	Vietnam	Community development	10	2^d^
2005	Niewöhner et al. [65]	Max-Delbrueck-Center for Molecular Medicine (MDC)	Germany	Relationship between biomedicine and economy in Germany	10	4
2012	Retèl et al. [64]	Netherlands Cancer Institute	Netherlands	Developments in technology assessment (e.g. clinical implementation of the 70-gene signature for breast cancer)	15	10 reduced to 5
2012	Rhea & Bettles [77]	Academy of Nutrition & Dietetics	US	Dietetics workforce supply and demand	10	4
2006	Rydström & Törnberg [58]	Karolinska Institute	Sweden	External influences on cervical cancer incidence and mortality	n.s.^c^	8
2011	Suk & Semenza [75]	European Centre for Disease Prevention and Control (ECDC)	Europe (Sweden)	Future infectious disease threats to Europe	10	8
2014	Vollmar et al. [21]	German Center for Neuro-degenerative Diseases (DZNE)	Germany	Health care for people with dementia in Germany	20	5
2008	Wiek et al. [66]	Institute for Environmental Decisions (IED)	Switzerland	Possible future developments of nanotechnology in Switzerland	10	5

^a^ *IAF* Institute for Alternative Futures, ^b^ *STG* Steering Committee on Future Health Scenarios, ^c^ *n.s.* not specified ^d^“the outputs were limited to the best and worst case scenarios” [30]

### Main topics

The main topics of the scenario projects differed in many ways. Most of them addressed disease related issues (*n* = 9), led by mental health and dementia (*n* = 4) [13, 21, 29, 69, 70] and cancer (*n* = 3) [58, 64, 74]. Only one scenario project each reported on cardio-vascular diseases [73] and infectious diseases [75].

Five scenario projects dealt with public health issues on an organizational level [24, 30, 53, 61, 76] and five on the labor market of different health care professionals [16, 23, 55, 60, 77], with two of them focusing on the pharmacy profession [16, 60].

In addition, four projects dealt with health care ‘in general’ [22, 63, 72, 78], four with other technology developments [57, 62, 64, 66], and an additional four with the field of biotechnology and personalized medicine [29, 59, 65, 71].

Three projects were concerned with the pharmaceutical industry and drug development [54, 56, 79]. We could identify only one scenario project each about the food industry [80], aging issues [73, 81], ‘recommendations’ for a developing country [18], academic medicine [82, 83], and the influence of climate change (on health) [84].

Eleven scenario projects (Table 1) were from North America, 25 from Europe (one of them with a topic about India [18]), one from Vietnam [30] and one had an international focus [82, 83]. More than half of the projects (*n* = 21) were published in the last ten years, eight between 1995 and 2004 and nine before 1995. Of these nine, six projects [67–70, 73, 74, 79, 81] were part of a national program in the Netherlands. During this program the study group for future scenarios in health care (STG/STC) developed several scenarios for certain health issues from the mid-1980s to the mid-1990s for the Dutch government [48, 73, 85]. In this review we considered (as mentioned in our inclusion/exclusion criteria) only those program projects which were published in scientific journals (*n* = 8) [67–70, 73, 74, 79, 81]. A full list of all topics covered by the STG/STC program can be found in the program description of Schreuder [48].

## Discussion

The scenario method has been used for a wide spectrum of strategic issues and different applications, starting with military planning in the 1960s [9–11, 52, 86, 87]. Despite its potential, use of the scenario method seems to have been published rarely in comparison to other methods such as the Delphi-technique, at least in the field of health care since the 1980s [40, 48, 88]. Our scoping review could identify 41 relevant publications in scientific journals representing 38 scenario projects. There were a lot of different perspectives as indicated by the wide range of participating institutions and experts (Table 1). The scenario projects addressed not only public health problems, but also strategic issues for business decisions (e.g. the future of the Danish food industry [80], the opportunities of nanotechnology in Switzerland [66], or the relationship between biomedicine and the economy in Germany [65]). One project even came from the Swedish Defense Research Agency (FOI) with tailor-made scenarios for local adaptation to climate change [84]. Nevertheless, this project addressed (among other things) the effects of a heat wave on the health care sector, which is definitely an important topic for public health researchers. One project described three fictive scenarios as examples for the scenario method itself [24]. We decided to include this “project” because it addressed a relevant public health issue (“using a health department’s desire to address chronic disease prevention and control”) [24]. Most of the projects were developed after the year 2000 and addressed a wide range of topics, from regional institutional perspectives (e.g. local public health departments [53]) to global challenges (e.g. future infectious disease threats to Europe [75]). Many of the scenario projects in this review provide a framework for determining actions in research, as well as in public policy-making, e.g. it could be the basis for discussing a national dementia plan [21, 69, 70] or for developing a strategy to ‘revitalize’ academic medicine [82, 83]. None of the projects has been designated as unsuccessful by the authors, which could be either a sign of the method’s strength or of publication bias. In fact, Gregório and colleagues stated: “The use of scenario analysis in a strategic thinking process has demonstrated to be of value while planning for future resources and other policy issues” [16]. Several of these scenario projects were classified as helpful for strategic planning and also for enabling the incorporation of expert knowledge (the qualitative ‘human factor’) [13, 48, 53]. Additionally, several projects used quantitative approaches to calculate the scenarios [21, 23, 56]. The resulting scenarios were illustrated in many different ways or combinations (e.g. tables [82], text descriptions [78], pictures [83], or short stories called storylines [21]). Although there is no definite response to the question of how many scenarios are optimal in the scenario planning literature [87], three to five scenarios are considered appropriate by most of the researchers [86, 87]. This number also occurred most frequently in our review. Scenarios can be described as outlines of possible variations of the future [88], but do not describe comprehensive pictures of the future and do not claim to be complete or correct [10]. Sometimes doubts with respect to the reliability of the scenarios may arise because the methods are not clearly described [15, 54, 78, 82, 83, 89]. Compared to conventional methodological reporting, i.e. in clinical studies, the method in scenario projects should be described as precisely as possible due to the process-oriented character of scenario development. This includes the selection of the experts, the applied software tools, the use of additional literature sources, and also the method’s use in combination with other methods, like the Delphi technique [67–71].

### Limitations

Although this review is quite comprehensive with respect to the scenario method in the field of health or health care, there are some limitations (partly inherent to the scenario method, see below) which should be mentioned. First of all, despite our detailed search strategy, it was difficult to accurately identify appropriate scenario projects. This issue is summarized by Glenn’s statement: “scenario is the most abused term in futures research” [9]. Bishop and colleagues added: “even the most basic vocabulary is used every which way in this field [89]. We provided some examples of this issue in the Methods section (reasons for exclusion). Even when an article’s title seems to be clear, one cannot be certain that the article deals with a scenario project [31, 90]. Furthermore, there is no clear-cut scenario method. Unlike classical epidemiological research, many variants of the method exist and, according to our findings, are applied in various projects [9, 10, 48, 87, 88]. An additional difficulty is that these different variants of the method have not remained stable. For example, there has been a notable shift from quantitative to more qualitative or mixed method approaches, as evidenced by statements like “Scenario-projects are primarily simulation” [81], “scenario analysis is essentially a qualitative technique” [53], “we have used […], the qualitative scenario method, in order to assess and rank possible influencing factors” [58]. According to Glenn, “often projections are confused with scenarios” [9]. Another limitation is that only 11 publications were identified from searches in the databases used in this review. The remaining 30 publications were found by screening the reference lists, on the internet, and through recommendations from experts in the field. It was also apparent that only a few scenario projects have been published in scientific journals (by researchers), whereas a substantial number of project reports have been published as grey literature by government institutions [25, 91], non-government organizations [47, 92] or private (commercial/consulting) firms [92, 93]. So, it should be taken into consideration that some of the scenario projects have not been developed by scientific experts, but rather came from non-scientific institutions or clinical organizations (with little awareness of scientific research techniques) [55, 62, 72]). Additionally, some included scenario projects [21, 29] have also been published in more detailed reports [94] or books [95].

### Decision against reviewing grey literature

The “Pisa Declaration on Policy Development for Grey Literature Resources” [96], a Cochrane report [97], and the enhanced Arksey and O´Malley framework [27, 28] all recommend including grey literature to validate the results of a research-based literature search. However, Levac and colleagues also point out the cost-to-benefit ratio consideration: “Balancing breadth and comprehensiveness of the scoping study with feasibility of resources can be challenging” [28].

We decided not to include grey literature because of the following reasons:

- Firstly, although non-peer reviewed publications have the potential to provide valuable insights in this area, the quality of methods applied to data collection, analysis and interpretation may vary substantially [98]. We conducted an exploratory search on the internet before we used a systematic search approach. We randomly surveyed the reports and found a very heterogeneous quality. Although there were a few reports reflecting high scientific quality (e.g. [25, 99]) we also identified reports of lesser quality and reports with minimal to non-existent descriptions of the methods used [93]. Notably, one can be assumed that reports from commercial/consulting firms [92, 93] try to avoid detailed and transparent description of the scenario preparation process in order to protect proprietary data and the nature of their business model.
- Secondly, in order to confirm that our results could be replicated, we wanted to ensure that all studies had been subjected to some form of peer-review. In order to still allow a maximal amount of comprehensiveness, we conducted a systematic search for literature in the mentioned databases and also in the reference lists of the identified literature (backward tracking).
- Thirdly, there is still a lack of persistent identifiers and open standards of metadata for grey literature, which complicates the identification of relevant publications.
- Fourthly, the large number of existing scenario projects addressed in reports (e.g. [25, 91, 93, 94, 99–101]) made it impossible to handle a comprehensive search with our limited resources. Because of that, the effort associated with a comprehensive search for grey literature would be disproportionate to the resulting benefits. For example, the study group for future scenarios in health care (STG/STC) listed 29 published books for their program alone [48]. For this program we identified eight scenario project articles published in scientific journals [67–70, 73, 74, 79, 81].

For these reasons, we think a subsequent integration of grey literature would not have led to new and stronger results for our research aims.

### Limitations of the scenario method itself

The following limitations are inherent to the scenario method itself and should be considered also in health planning [9, 10, 48, 87, 88]. Firstly, creating explorative scenarios can be time-consuming and therefore cost-intensive, in particular because they tie up personnel resources [21]. However, the processes are scalable; a small group might be able to develop consistent scenarios, for example [58, 61]. Secondly, the quality of the scenarios depends greatly on the imagination, information basis and competency of the experts taking part [21, 102]. Thus, there is a potential risk of biased scenarios if experts are inclined to give preference to well-known developments and to reject any that seem too unorthodox; or in other words: opinion leaders who try to dominate a scenario group are counterproductive [64]. As a result, the selection of the experts is of considerable importance and should depend on the criteria applied to consensus processes and Delphi methods [64, 102]. But, as shown in the included scenario projects, it is possible to get usable conclusions with ‘ordinary’ persons acting as experts [29, 30, 65]. Thirdly, if the scenario developing process is not only narrative like some included projects [55, 58, 72], but also includes quantitative aspects by means of calculations [64, 81], the mathematical processes used to generate the scenarios may be plausible for scenario-natives but incomprehensible for non-experts [21]. Thus, unlike other methods (e.g. Monte-Carlo simulation), there is no standard gateway for the researcher to use common tools or packages. Fourthly, the selection of the key factors is the crucial point of each scenario analysis [9]. In principle, a systematic search for each selected key factor is desirable to generate evidence, but available resources would hardly allow this. Fifthly, scenarios are not forecasting the future as each step always entails subjective assessments and evaluations of abstract and complex facts. So, another threat might be the overestimation of the exactness of explorative scenarios. The sixth and final point seems to be critical for the acceptance of the scenario method as a scientific tool particularly in the field of public health. Even though the reporting of the more common Delphi technique has room for further improvement [5, 8], it seems that the variability in using and reporting the scenario method is much higher [10, 15, 87, 103]. The authors strongly believe that there is a need to improve the reporting of scenario projects, along the lines of a GRAMMS-like guideline which is used for mixed methods studies and recommended by the equator-network (www.equator-network.org) [104]. Proposed indicators as a result from this review are listed in Table 2. Only if the transparency required to reproduce the underlying evidence exists, will the scenario method be a useful tool for future health care planning and strategic public health decision-making [103].

**Table 2:** Proposed key methodological criteria to report in scenario projects

Criteria
Aim of the scenario project
• Does the word “scenario project (planning, approach…)” appear in the title along with the topic of primary interest?
• Is the topic of interest clearly described?
• What are the proposed implications?
• Are the target groups and/or stakeholders specified?
• Is there a clear time horizon?
Framework of the scenario project
• Are the preconditions and presuppositions well described?
• Is the process of developing the areas of influence, key factors, and future projections adequately described?
• Who is involved (description of scenario development team and participants/experts)?
• Is the background of participants/experts clear?
• How will participants/experts be selected or excluded?
Methodological approach of the scenario project
• Is the specific scenario technique used (e.g. only narrative, consistence analysis, cross-impact analysis) well described?
• If any, is the mathematical approach well described?
• How is the mathematical approach transformed/implemented in software (if applicable)?
• Is there any combination with other methods like the Delphi technique?
• Is the presentation of the scenario development process adequate?
• Are the scenarios presented in a sound manner (to the specified target groups/stakeholders)?
Impact of the scenario project
• Are there any recommendations for different target groups/stakeholders?
• What are the next steps after the scenario project?

## Conclusions

In recent years, more scenario projects relating to health and health care have been published in scientific journals than ever before. This review provides a comprehensive overview of the use of the scenario method in the field of public health and health policy research. The scenario method has been classified as most helpful for strategic issues by several authors of the projects. However, there is no ‘one” scenario method. There is a wide spectrum of strategic aims covered by heterogeneous variants of the scenario method.

To establish the scientific use of scenario methods, uniform qualitative reporting would be useful, based on the GRAMMS criteria, for example [104].

## Additional files

Additional file 1: **Supplementary data 1: Search strategies.** (DOCX 17 kb)
Additional file 2: **Supplementary data 2: Inclusion and exclusion criteria (see also Fig.** 1**).** (DOCX 16 kb)
