# Supplementary material for: Using the scenario method in the context of health and health care – a scoping review
Source: BMC Med Res Methodol. 2015 Oct 16;15:89. doi: 10.1186/s12874-015-0083-1 (PMC4609149; doi:10.1186/s12874-015-0083-1)
Supplement: Additional file 1: — Supplementary data 1: Search strategies. (DOCX 17 kb) [file 12874_2015_83_MOESM1_ESM.docx]

**Additional file 1 Supplementary data 1: Search strategies**

***Databases: Medline; Embase; PsycInfo; Eric; The Cochrane Library
Date: inception to 2013-01-30 & 2013-02-01 to 2013-10-17***

1 scenario* technique*.tw,ot. (7)

2 Scenario* model*.tw,ot. (71)

3 scenario* process*.tw,ot. (2)

4 scenario* project*.tw,ot. (29)

5 scenario* method*.tw,ot. (23)

6 or/1-5 (132)

7 health.tw,ot. (896341)

8 exp "Delivery of Health Care"/ (729346) (in Embase: exp Health care/)

9 7 or 8 (1400095)

10 6 and 9 (23)

***Database: Scopus / Date: inception to 2013-01-29 & 2013-01-30 to 2013-10-17***

(((TITLE-ABS-KEY("scenario* technique*") OR TITLE-ABS-KEY("scenario* process*") OR TITLE-ABS-KEY("scenario* project*") OR TITLE-ABS-KEY("scenario-technique*") OR TITLE-ABS-KEY("scenario-process*") OR TITLE-ABS-KEY("scenario-project*") OR TITLE-ABS-KEY("scenario* method*") OR TITLE-ABS-KEY("scenario* model*") OR TITLE-ABS-KEY("scenario-method*") OR TITLE-ABS-KEY("scenario-model*"))) AND (TITLE-ABS-KEY(health)))

***Database: Web of Science / Date: inception to 2013-01-29 & 2013-01-30 to 2013-10-17***

Topic=("scenario* technique*") OR Topic=("Scenario* process*") OR Topic=("scenario* project*") OR Topic=("scenario-technique*") OR Topic=("scenario-process*") OR Topic=("scenario-project*") OR Topic=("scenario* method*") OR Topic=("scenario-method*") OR Topic=("scenario model*") OR Topic=("scenario-model*")
Refined by: Research Areas=(PSYCHOLOGY OR GENERAL INTERNAL MEDICINE OR HEALTH CARE SCIENCES SERVICES OR SOCIAL SCIENCES OTHER TOPICS)

***Database: CINAHL / Date: inception to 2013-03-26***

1. Scenario technique in health care research:

1 TX scenario* technique* OR TX scenario* process* OR TX scenario* project* OR TX scenario- technique* OR TX scenario* model* OR TX scenario* method*

2 TX health

3 S1 AND S2

***Other sources: Google scholar / Date: 2013-12-18***

- health care scenarios

**Articles offered by Pubmed */ Date: 2015-05-15 (post hoc inclusion)***

- related to Vollmar HC et al. 2014 / Gregório J et al. 2014 / Nguyen V et al. 2014

***Additionally***

- text tracking (backwards) of all included papers
