# Supplementary material for: Using the scenario method in the context of health and health care – a scoping review
Source: BMC Med Res Methodol. 2015 Oct 16;15:89. doi: 10.1186/s12874-015-0083-1 (PMC4609149; doi:10.1186/s12874-015-0083-1)
Supplement: Additional file 2: — Supplementary data 2: Inclusion and exclusion criteria (see also Fig. 1). (DOCX 16 kb) [file 12874_2015_83_MOESM2_ESM.docx]

**Additional file 2 Supplementary data 2: Inclusion and exclusion criteria (see also Fig. 1)**

***Inclusion criteria***

- for title and abstract screening:
  - Does the title or abstract indicate a scenario project?
- for 1st level full text screening:
  - Is the term “scenario” used in a way which does not meet the exclusion criteria of the review?
- for 2nd level full text screening:
  - Does the paper describe a scenario project (or is it “only” a methods or descriptive program article)?
- for all stages:
  - Is the topic of the paper related to health or health care?

***Exclusion criteria***

Exclusion criteria (for all stages) were:

- the term ‘scenario’ was used only to refer to a possible (future) event
- 'scenarios' in epidemiology when used only as projections (e.g. 'population ageing', defined as an increase in the percentage of elderly persons in the population)
- 'scenarios' which were 'pure' simulation modeling
- 'scenarios' which were used only to support shared decision-making (e.g. determination of patient preferences)
- 'scenarios' in microbiology or genetics
- description of the scenario method itself (without a concrete project)
- publications unrelated to health or health care
- abstracts only (no full-text available or full-text already included in our review)
- grey literature (e.g. reports) without publication in a scientific journal
